# Influence of new qualitative productivity forces on quality-effective change in China’s Yangtze river economic belt sports industry: The mediating effect of industry–University–Research Cooperation

**DOI:** 10.1371/journal.pone.0319366

**Published:** 2025-02-25

**Authors:** Xiuqin Chen, Songzhong Ye

**Affiliations:** Sports Industry Development Research Center, Fujian Jiangxia University, Fuzhou, China; Shenzhen University, CHINA

## Abstract

Due to the lack of empirical research on the influence of new qualitative productivity forces on quality-effective change in the sports industry, entropy-weighted threshold-based estimation (TOPSIS) is used based on panel data for 11 provinces and cities in China’s Yangtze River Economic Belt from 2016–2022. The results are used to measure the levels of new qualitative productivity forces and quality-effective change in the sports industry and to analyze the relationships among these factors and industry–university–research cooperation through a bidirectional fixed effects model and a mediating effects model. The study reveals that new qualitative productivity forces have a significant positive effect on quality-effective change in the sports industry that is heterogeneous by region. Industry–university–research cooperation plays a partially intermediary role in promoting quality-effective change in the sports industry through new qualitative productivity forces. In this context, we propose the following measures: focus on cultivating new-quality industries and strengthening their application in the sports industry, emphasize the intermediary effect of industry–university–research cooperation to promote quality-effective change in the sports industry, formulate strategies according to local conditions, and focus on the development of new qualitative productivity forces in sports.

## Introduction

New qualitative productivity forces are the result of revolutionary technological breakthroughs and the collection of many emerging technologies that play a sustained role in this context. The process by which such productivity is generated is often accompanied by transforming and upgrading traditional industries while cultivating and growing new industries. Economic development refers to the organic unity of quality and quantity to ensure that the economy continues to engage in high-quality development in terms of both quality and efficiency; in this context, the important role of new qualitative productivity forces must be prioritized [1]. At the third plenary session of the 20th Central Committee of the Communist Party of China, it was noted that the nation should further promote both the real and the digital economy through the in-depth integration of the system and the development of new qualitative productivity forces in accordance with local conditions to accelerate the cultivation of a new form of kinetic energy. As an important component of China’s real economy, the sports industry, after years of rapid development, is currently in urgent need of the transformation of its development mode. To complete the transformation and upgrading of the traditional sports industry, it is necessary to take active advantage of new-quality productivity and inject new vitality and momentum into the sports industry through multi-dimensional strategies such as pioneering scientific and technological innovation, strengthening support for talent, promoting green development, optimizing the industrial structure, and upgrading the level of the industrial chain. Therefore, an urgent research problem has arisen, namely, how to effectively integrate new qualitative productivity forces and inject new vitality into the sports industry through multidimensional measures such as scientific and technological innovation leadership, talent resource optimization, green development strategies, industrial structure upgrading and industry chain-level enhancement. Therefore, this paper focuses on elaborating and analyzing the relationships among new qualitative productivity forces, industry–university–research cooperation and quality change in the sports industry and performs research according to the logical pathway of suggesting, analyzing, and solving problems. Specifically, this paper asks why we should carry out research on the impact of new qualitative productivity forces on quality change in the sports industry in China. Then, it discusses the relationship among new qualitative productivity forces, industry–university–research cooperation and quality change in the sports industry. Finally, the paper presents ways of promoting quality change in the sports industry through new qualitative productivity forces and industry–university–research cooperation to promote quality and efficiency change in the sports industry.

## Literature review

At present, academic research on new qualitative productivity forces and quality and efficiency change in the sports industry has focused mainly on the following three aspects. The first is in relation to new qualitative productivity forces. Scholars have focused on new qualitative productivity forces in terms of theoretical connotations, development characteristics, formation logic and practice paths [2–10]. New qualitative productivity forces is the qualitative change in productivity required for high-quality development of the economy, the rapid formation of which depends on a high level of scientific and technological innovation and self-reliance. Second, research has focused on the relationships between new qualitative productivity forces, on the one hand, and industrial quality and efficiency change, on the other hand. Ren Baoping and Wang Ziyue (2023), Shen Kunrong et al. (2024), and Jia Ruoxiang et al. (2024) believe that new qualitative productivity forces accelerate the change of production mode and promotes high-quality development [11–13]. Xu Zheng et al. (2023) further noted that the cultivation and growth of new qualitative productivity forces can help achieve the goal of development, enhance power, improve the structure, expand the content and optimize the factors of industry [14]. Third, research is needed on the relationship between new qualitative productivity forces and the qualitative change in the sports industry. In terms of qualitative research, Luo Xiwen and Bao Mingxiao (2024), Pan Kefan and Shen Keyin (2024), Yang Fengying et al. (2024), and Shizi Lizhen et al. (2024) explored the driving mechanism, internal mechanism, difficulties in promotion and path selection of new qualitative productivity forces to promote the qualitative change in the sports industry [15–18] These results provide useful references for this study in terms of theoretical analyses and research hypotheses. No empirical research has yet been carried out on whether new qualitative productivity forces can directly promote quality change in the sports industry, but some scholars have begun to empirically analyze the impact of the digital economy on quality change in the sports industry and the transmission path. For example, Su Weizhou et al. (2024) empirically analyzed the role of the digital economy in promoting quality change in the sports industry and the role of industrial structure and scientific and technological innovation as intermediary and moderating variables through a fixed effects model, a mediation model and a threshold model [19]. Guo Wei et al. (2024) used a panel regression model to empirically test the effect and mechanism of the digital economy on quality change in the sports industry by constructing an evaluation index system of the quality change level of the industry [20]. Through rigorous empirical methods and scientific evaluation systems, scholars have enriched the theoretical research on the driving role of the digital economy in related fields, improved the evaluation system of the sports industry and its influencing factors, and provided important references for this study in terms of framework construction and method selection.

This overview shows that academic research on the impact of new qualitative productivity forces on quality-effective change in the sports industry has gradually expanded, but studies have explored the mechanism of how new qualitative productivity forces promotes quality-effective change in the sports industry, and no studies have empirically investigated the impact of new qualitative productivity forces on quality-effective change in the sports industry. Therefore, two perspectives are considered in this paper: the specific impact of new qualitative productivity forces on the quality-effective change in the sports industry, with the Yangtze River Economic Belt selected as an example to analyze the heterogeneity of different regions, and the mediating effect of industry–university–research cooperation on the influence of new qualitative productivity forces on the quality-effective change in the sports industry. This approach can enhance the overall understanding of the relationship between new qualitative productivity forces and quality-effective change in the sports industry and provide theoretical support and a decision-making reference for the formulation of more precise and effective policy measures.

## Theoretical analysis

### Analysis of the direct effects of new qualitative productivity forces

New qualitative productivity forces are based on improvements related to laborers, labor materials, labor objects and their optimal combination “to promote quality with the new” and drive quality-effective change with innovation. Accelerating new qualitative productivity forces can help optimize the structure of the industrial system, expand the scope of industrial development, enable quality-effective change in the economy [14], and promote quality-effective change in the sports industry [16].

On the one hand, new qualitative productivity forces can help optimize the development structure of the sports industry. The key aim of quality-effective change in the sports industry is to improve the quality of production factors and total factor productivity as well as to improve the overall effectiveness of the industrial system comprehensively. As a contemporary, advanced mode of productivity, the core symbol of new qualitative productivity forces is the improvement of total factor productivity [21]. Accelerating the development of new qualitative productivity forces can improve the overall efficiency of the sports industry and optimize its structure. After years of development, China’s sports industry has completed its transformation from a traditional sports manufacturing industry to a sports service and manufacturing industry, and the corresponding industrial system has become increasingly complex; namely, it no longer refers to a simple amalgamation of several industrial categories [22]. Because new modes, forms, and products continue to emerge in the sports industry, the cross-border integration of cutting-edge science and technology has become increasingly prominent, and the corresponding technical barriers have gradually disappeared. Therefore, in the digital era, taking full advantage of technological innovation and digital transformation can effectively promote benign interactions among the links in the sports industry chain, enterprise, regions and subsystems, thus promoting the quality and efficiency of the sports industry and sustainable development.

However, new qualitative productivity forces can help expand the content of sports industry development. The goals of quality-effective change in the sports industry are to achieve better quality and efficiency as well as to promote the continuous synergistic development of the scale of the sports industry, the construction of sports culture, and a social security system for sports while meeting people’s needs for a better life. New qualitative productivity forces can promote the growth of total economic volume by optimizing the allocation of production factors and enhancing production efficiency [23]; enhance the quality of factors in the cultural industry by expanding the object of labor, establishing high-tech processes for labor and shaping high-quality producers [24]; and force the social security system to adapt to the changes entailed by the reduction in jobs in traditional industries and the increase in jobs in new industries with the goal of meeting people’s growing social security needs. In terms of the sports industry scale, new qualitative productivity forces can enhance the overall effectiveness of the sports industry and optimize its structure; thus, new qualitative productivity forces can promote a significant increase in the total output and value added of the industry. In the construction of a sports culture, new qualitative productivity forces expand the application scenarios of digital technology in the field; promote innovation within the content-based sports culture industry; generate new industry forms such as digital sports art, performance, and entertainment; and promote the prosperous development of sports culture. In terms of sports social security, digital productivity can optimize the allocation of social security resources; use big data to identify athletes’ social security needs accurately, including career transition, psychological counseling and medical rehabilitation; and support efforts to improve the sports social security system.

Therefore, Hypothesis 1 is proposed as follows: new qualitative productivity forces can directly promote quality-effective change in the sports industry.

### Analysis of the mediating effect of industry–university–resea

Industry–university–research cooperation has an important mediating effect that can promote the ability of new qualitative productivity forces to inspire quality-effective change in the sports industry. Industry–university–research cooperation is a highly collaborative, deeply integrated and innovative cooperation mode that features a clear division of labor. It combines the innovation resources of universities and research institutions with market demand and the industrialization capacity of enterprise with the goal of generating strong innovation synergy, thus accelerating revolutionary technological breakthroughs and talent resource sharing as well as promoting quality-effective change in the sports industry. First, industry–university–research cooperation can effectively shorten the application cycle of technological innovation for new qualitative productivity forces. Colleges, universities and scientific research institutions continually produce new theoretical achievements and achieve technological breakthroughs through scientific research activities, and these achievements can rapidly enhance the actual productivity of enterprises through industry–university–research cooperative platforms; additionally, enterprises use their market knowledge and industrialization ability to apply technological innovations in actual production, thus promoting technological advancement and product upgrading in the sports industry. The cooperation platform effectively shortens the cycle from technological innovation to industrial application and improves the innovation efficiency and market competitiveness of the sports industry. Second, industry–university–research cooperation has an important intermediary effect on talent cultivation and resource sharing. Through industry–university–research cooperation, universities and scientific research institutions can provide students with valuable practice opportunities and employment channels and cultivate more complex talent with high levels of innovation and practical experience in the sports industry. Additionally, enterprises can carry out staff training and technical exchanges with the aid of resources from universities and scientific research institutions to improve the professional accomplishments and innovation levels of their staff. Industry–university–research cooperation can also enhance the sharing of resources such as scientific research equipment, technical information, and market data; optimize resource allocation; and improve the efficiency of innovation. Finally, industry–university–research cooperation overcomes traditional barriers to cooperation and further improves this mediating effect. Industry–university–research cooperation includes not only traditional technology transfer, joint R&D and other processes but also new types of cooperation modes, such as the coconstruction of innovation platforms and the establishment of strategic alliances for innovation. Diversified cooperation modes can better adapt to the needs and characteristics of different enterprises and help achieve the in-depth integration of industry, university, and research groups in a win‒win scenario for all parties.

Therefore, Hypothesis 2 is proposed as follows: new qualitative productivity forces can indirectly promote quality-effective change in the sports industry through industry–university–research cooperation.

### Analysis of regional heterogeneity in the push effect

As a result of the different natural resources, geographical conditions and development strategies that characterize the provinces and cities in the Yangtze River Basin, obvious economic differences exist among the upstream, midstream, and downstream regions with respect to the development process. The imbalance of regional economic development affects the industrial development of each region and may likewise affect new qualitative productivity forces to drive quality-effective change in the sports industry. Therefore, the differences in new qualitative productivity forces that drive quality-effective change in the sports industry in different regions are analyzed in further detail. First, the provinces and cities of Shanghai, Jiangsu, and Zhejiang in the downstream region have historically led China’s economic development. They possess a robust market system, a plentiful supply of human resources and capital, and a strong capacity to cultivate a new economy and new forms of business. These factors are conducive to the digital transformation of the sports industry. Second, Anhui and Hubei in the midstream region boast convenient transportation, large population bases, superior geographic locations, and a relatively balanced industrial structure; all these factors establish a good external environment for the digitization of the sports industry. However, Hunan and Jiangxi have weak capital and technological strength and low levels of economic development, which is not conducive to promoting the transformation and upgrading of the sports industry through innovation and research and development. Finally, Sichuan and Chongqing in the upstream region have a wealth of natural resources, and their development of new industries is progressing well. The industrial structure is undergoing continual adjustment, providing a resource guarantee and expanding the scope for the development of new productivity.

Therefore, Hypothesis 3 is proposed as follows: there is regional heterogeneity in the effect of new qualitative productivity forces on the promotion of quality-effective change in the sports industry.

## Variable settings and data sources

### Variable settings

#### Dependent Variables.

The construction of a quality‒efficiency change evaluation system for the sports industry is important for monitoring the process of improving quality and efficiency and transforming and upgrading the sports industry [25]. The core elements of quality-effective change in the sports industry include development momentum; industrial efficiency; the industrial structure, scale, and foundation; and production efficiency [26]. Owing to the late emergence of China’s sports industry, data accumulation is relatively limited, some regions lack statistics on sports industry-related data [27]. Therefore, based on the specific characteristics of the sports industry and the availability of data, this paper draws on the method of Su Weizhou et al. (2024) [19] and refers to the relevant indicators constructed by Li Rugraphics et al. (2020) and Kang Lu et al. (2022) [26,28]. The industrial scale, foundation, and structure are selected to measure the level of quality-effective change in the sports industry, and the specific indicators are shown in Table 1.

**Table 1:** Evaluation indicators for quality-effective change in the sports industry.

Dimension	Factor layer	Measurement indicators (units)	Weights	Causality
Industrial scale	Total outputs	Y1 Total output of the sports industry (billion CNY)	0.2070	+
	Value added	Y2 Value added of the sports industry (billion CNY)	0.1905	+
Industrial foundation	Space provision	Y3 Area of sports space per capita (m^2^)	0.1722	+
	Financial investment	Y4 Expenditures of the Sports Lottery Community Chest (billion CNY)	0.1609	+
Industrial structure	Economic efficiency	Y5 Total output of the sports industry as a percentage of GDP (%)	0.0990	+
	Rationalization	Y6 Value added by the sports industry as a percentage of GDP (%)	0.1704	+

Note: “+” indicates a positive indicator; “-” indicates a negative indicator.

#### Independent variables.

Evaluating new qualitative productivity forces has become an urgent academic issue. Different scholars and research teams may propose their own evaluation indicator systems and construction methods based on different theoretical frameworks, research conclusions and data. In this context, the most representative evaluation systems can be divided into two categories: (1) comprehensive evaluation indices based on the three dimensions of laborers, labor objects and the means of production and (2) evaluation systems based on the three dimensions of scientific and technological productivity, green productivity, and digital productivity [29]. Based on the availability of relevant data, an evaluation system is established to assess the level of new qualitative productivity forces based on the indices proposed by Jue Wang et al. (2024), Yi Liu et al. (2024), and Yuxin Ren et al. (2024) [30–32]; notably, evaluation indices are established from seven dimensions: the three major components of productivity, namely, laborer attitude, quality, and productivity; the new-quality industry; the ecological environment; intangible labor factors; and material labor factors. The specific measurement indices are shown in Table 2.

**Table 2:** Indicators for evaluating new qualitative productivity forces.

Dimension	Target level	Factor layer	Measurement indicators (units)	Weights	Causality
laborer	Attitude	Spirit of innovation	X1 Full-time equivalent of R&D personnel (in man–years)	0.0619	+
		Enterprising spirit	X2 Number of new start-ups per 100 people (households)	0.0293	+
	Quality	Educational attainment	X3 Average years of schooling per capita (in years)	0.0209	+
		Cumulative knowledge	X4 Number of students in school/total population (%)	0.0425	+
		Funds for incubation	X5 Expenditure on education/total fiscal expenditure (%)	0.0313	+
	Productivity	Employment structure	X6 Tertiary/total employment (%)	0.0351	+
		Economic output	X7 GDP/total population (%)	0.0417	+
		Economic income	X8 Average wage of employed workers (in Yuan)	0.0312	+
target audience	New-quality industry	Emerging industry	X9 Technology market turnover (billion CNY)	0.0524	+
		Future industries	X10 Number of robots/total population (%)	0.0368	+
	Ecological environment	Green production	X11 Number of green patent applications/patent applications (%)	0.0544	+
			X12 Sulfur dioxide emissions/GDP (%)	0.0717	–
		Green ecology	X13 Forest cover (%)	0.0423	+
			X14 Environmental protection expenditure/public finance expenditure (%)	0.0644	+
labor resources	Intangible labor resources	Technological innovation	X15 Number of patents granted/total population (%)	0.0505	+
			X16 Expenditure on new product development/GDP (%)	0.0540	+
		Level of digitization	X17 Digital economy index (‒)	0.0500	+
			X18 Enterprise digitization level (‒)	0.0554	+
	Material labor resources	Infrastructure	X19 Fiber length (in km.)	0.0490	+
			X20 Internet broadband access ports per capita (individual)	0.0812	+
		Energy consumption	X21 Energy consumption/GDP (%)	0.0250	–
			X22 Renewable energy electricity consumption/electricity consumption of society as a whole (%)	0.0189	+

Note: “+” indicates a positive indicator; “-” indicates a negative indicator

#### Mediating variable.

Innovation is key to quality-effective change in the sports industry, and the effective transformation of scientific and technological achievements is an important means of opening the innovation chain. Industry–university–research cooperation has accelerated the transformation and application of scientific and technological achievements and is key for the implementation of innovation-driven development strategies. Therefore, industry–university–research cooperation is selected as the mediating variable in this study, drawing on Zhang Fan et al. (2022) [33]. To assess the intensity of industry–university–research cooperation, the ratio of enterprise funding sources for internal expenditures in each region given the available research and experimental development (R&D) funds to the total internal expenditures of R&D funds is used to indicate the intensity of the linkages among industry–university–research entities. When the ratio is higher, an enterprise’s investment in R&D activities is higher, as is the level of cooperation with academia and research, thus promoting technological innovation and industrial upgrading.

#### Control variables.

Quality-effective change in the sports industry is a complex and dynamic process involving multiple fields—such as the economy, society and technology—which are jointly determined by a variety of internal and external factors. Therefore, based on existing research results, the levels of economic development (Pgdp) [34], marketization (Mark) [35], and technological innovation (Tec) [36]; the industrial structure (Ind) [37]; the degree of government intervention (Gover) [38]; and the level of openness to the outside world (Open) [39] are selected as the control variables that may promote quality-effective change in the sports industry. The specific indicators are defined as the logarithm of per capita GDP, the logarithm of the marketization index, the logarithm of the patent authorization index, the added value of the tertiary industry as a proportion of GDP, the proportion of local general public budget expenditures as a proportion of GDP, and the total amount of imports and exports as a proportion of GDP.

### Data sources

As China’s statistical system for the sports industry is not yet sound, it is impossible to obtain complete data regarding the total output and added value of the sports industry in 31 provinces and cities across the country; thus, 11 provinces and cities along the Yangtze River from 2016–2022 are selected as samples for this research. In this context, the sports industry data are obtained mainly from the website of the State General Administration of Sports, the websites of the sports bureaus associated with each province and city, and the statistical yearbook of sports in China; the data concerning new qualitative productivity forces and other variables are obtained mainly from the statistical yearbooks for each province and city, the China Statistical Yearbook, the China Scientific and Technological Statistical Yearbook, the CEIC China Statistical Database, the China Energy Statistical Yearbook, and the China Environmental Statistical Yearbook. Most of the indices were based on secondary calculations with the original data, and the missing data for a few years in individual provinces and cities were obtained via interpolation and the moving average method.

## Method selection and model construction

### Entropy-weighted TOPSIS method

In the evaluation of new qualitative productivity forces and quality-effective change in the sports industry, because many factors are involved, it is often difficult for a single indicator to comprehensively and accurately reflect the real situation; thus, the use of a comprehensive multi-indicator evaluation method is particularly important. As an effective multicriteria decision-making method, the entropy-weighted TOPSIS method can be implemented with raw data and can accurately reflect the gaps among evaluation schemes, providing an effective solution for resolving complex problems. The method has been widely used in quality-effective change evaluations in the economic, agricultural, logistics and tourism fields [40–43]. Therefore, in this paper, the entropy weight TOPSIS method is used to measure the composite evaluation index of the two dependent and independent variables. The specific calculation steps are as follows.

In the first step, given that the unit and scale of each index are different, which could influence the statistical results, the data are intervalized and normalized before the weight of each index is calculated.


$$Y_{ij}=\frac{X_{ij}-\min X_{j}}{\max X_{j}-\min X_{j}}$$ (1)

$$Y_{ij}=\frac{\max X_{j}-X_{ij}}{\max X_{j}-\min X_{j}}$$ (2)

Equations (1) and (2) are used for positive and negative indicator data in the raw data, respectively, where *X*_*ij*_ is the original indicator value; *Y*_*ij*_ is the intervalized indicator value; *i* and *j* represent the province and indicator, respectively; and *minX*_*ij*_ and *maxX*_*ij*_ are the extremes of the *j*th indicator.

In the second step, the indicator weights, entropy values and final weights are calculated as follows:


$$b_{ij}=\frac{Y_{ij}}{\sum_{i=1}^{n}Y_{ij}}$$ (3)

$$S_{j}=\frac{1}{\ln n}\times \sum_{i=\;1}^{n}\left[b_{ij}\times \ln\left(b_{ij}\right)\right]$$ (4)

$$Q_{j}=\frac{1-S_{j}}{\sum_{j=1}^{m}1-S_{j}}$$ (5)

where *i* and *j* represent the province and the indicator, respectively; while *Q*_*j*_ is the final weight of the indicator; *S*_*j*_ is the entropy value of the indicator; and *b*_*ij*_ is the share of indicators.

In the third step, the composite index value is calculated via the TOPSIS method:


$$\text{Weighting matrix: }w={\left(w_{ij}\right)}_{m\times n},w_{ij}=Q_{j}\times Y_{ij}$$ (6)

$$\text{Optimal solution:}Z_{j}^{+}=\max\left(w_{ij}\right);Z_{j}^{-}=\min\left(w_{ij}\right)$$ (7)

$$\text{European distance:}D_{i}^{+}=\sqrt{\sum_{j=1}^{m}{\left(Z_{j}^{+}-w_{ij}\right)}^{2}};D_{i}^{-}=\sqrt{\sum_{j=1}^{m}{\left(Z_{j}^{-}-w_{ij}\right)}^{2}}$$ (8)

$$\text{Composite index:}\;C_{i}=\frac{D_{i}^{-}}{D_{i}^{+}+D_{i}^{-}}$$ (9)

### Model construction

A two-way fixed effects model is constructed to test H1. Two-way fixed effects models are commonly used for cases with panel data and can provide accurate estimation results. The model is shown below:


$$Shd_{it}=\alpha_{0}+a_{1}Nqp_{it}+\beta_{1}X_{it}+u_{i}+\theta_{t}+\varepsilon_{it}$$ (10)

where *Shd*_*it*_ is an explanatory variable, the level of quality-effective change in the sports industry; *Nqp*_*it*_ is an explanatory variable, the level of new qualitative productivity forces; *X*_*it*_ is a control variable; *i* and *t* represent the province, city, and time, respectively; *ε*_*it*_ is a random perturbation term; and *u*_*i*_ and *θ*_*t*_ are the year and province or city fixed effects, respectively.

A mediated effects model is constructed to test H2. Mediating effects models can separate direct and indirect effects, avoiding the limitations of single effect analysis and helping to accurately assess the effects of different factors on quality-effective change in the sports industry. The model is shown below:


$$Shd_{it}=\alpha_{0}+a_{1}Nqp_{it}+a_{2}X_{it}+u_{i}+\theta_{t}+\varepsilon_{it}$$ (11)

$$Cw_{it}=\beta_{0}+\beta_{1}Nqp_{it}+\beta_{2}X_{it}+u_{i}+\theta_{t}+\varepsilon_{it}$$ (12)

$$Shd_{it}=\gamma_{0}+\gamma_{1}Nqp_{it}+\gamma_{2}Cw_{it}+\gamma_{3}X_{it}+u_{i}+\theta_{t}+\varepsilon_{it}$$ (13)

where *Cw*_*it*_ is the mediating variable industry–university–research cooperation, and the other variables are the same as those in Equations (10).

Model (10) is used to test H3. The two-way fixed effects model controls for both individual (regional) and time fixed effects, which helps eliminate the negative effects of unobservable factors on the estimation results due to regional or temporal differences. In this paper, we reveal the heterogeneity of the driving effect in different regions by comparing the magnitude and significance level of the estimated values of new qualitative productivity forces in the upstream, midstream, and downstream regions of the Yangtze River.

## Empirical results and analysis

### Benchmark regression analysis

To identify evidence indicating that new qualitative productivity forces can directly drive quality-effective change in the sports industry, this paper constructs a panel model. The first step in this process involves selecting the optimal model from the three developed models. As shown in Table 3, the conclusions of both the F test and the Hausman test indicate that the FE model is superior to both the POOL model and the RE model. Therefore, the FE model is selected as the optimal model with which to analyze the relationship between the quality-effective change level of the sports industry and the corresponding influencing factors.

**Table 3:** Optimal model test results (n = 77).

Type of test	Comparison model	Test value	Test conclusion
F test	FE and POOL modeling	F(10,59) = 14.678, p = 0.000 < 0.05	FE
BP test	RE and POOL modeling	X^2^(1) = 43.275, p = 0.000 < 0.05	RE
Hausman test	FE and RE modeling	X^2^(6) = 385.210, p = 0.000 < 0.05	FE

The benchmark regression results presented in Table 4—particularly Result (1), in which no other control variables are added—reveal that the regression coefficient value of the development level of new qualitative productivity forces is 1.403, and the significance level reaches 0.01 (t = 8.822, p < 0.01), thus indicating that new qualitative productivity forces have a direct driving effect on the quality-effective change in the sports industry. Regression Result (2) was then obtained by adding six control variables to Result (1); the regression coefficient value of the development level of new qualitative productivity forces, as shown in in Result (2) is 1.015, while the level of significance remains 0.01, thus continuing to suggest a significant positive effect. Accordingly, H1 is valid; i.e., new qualitative productivity forces can directly promote quality-effective change in the sports industry. Result (2) demonstrates that the regression coefficients of the variables of economic development, marketization, technological innovation, industrial structure and opening up to the outside world are positive, and all of these figures are significant at the 5% and 1% levels. This finding indicates that the growth of per capita GDP, the improvement of the marketization level, the increase in patent authorization, the increase in the added value of the tertiary industry, and the growth of the total amount of imports and exports can promote quality-effective change in and development of the sports industry. Notably, the regression coefficient of government intervention is negative and significant at the 0.01 level (t = ‒4.483, p < 0.01), thus indicating that the degree of government intervention has a significant negative effect on quality-effective change in the sports industry and that excessive government intervention may interfere with the natural development of the market, which is conducive to neither quality-effective change in nor the healthy development of the sports industry.

**Table 4:** Benchmark regression.

Variant	Shd					
(1)	(2)	(3)	(4)	(5)	(6)
Nqp	1.403** (8.822)	1.015** (5.921)	1.663** (6.564)	1.402** (4.825)	0.826** (2.747)	1.242** (3.920)
Pgdp		0.198** (5.922)		0.298** (5.264)		0.214** (4.004)
Mark		0.318** (4.589)		0.197 (1.454)		0.779** (7.659)
Tec		0.066** (3.685)		0.097** (3.263)		0.131** (4.753)
Ind		0.430* (2.406)		0.555 (1.228)		0.205 (0.648)
Gover		‒1.326** (‒4.483)		‒1.172 (‒1.452)		‒1.751** (‒4.648)
Open		1.076** (3.825)		‒0.651 (‒1.067)		0.211* (2.394)
Constant	‒0.191* (‒2.511)	‒2.734** (‒3.104)	‒0.301** (‒2.907)	‒3.238** (‒5.716)	‒0.423** (‒3.105)	‒2.579** (‒4.681)
R^2^	0.335	0.369	0.672	0.831	0.306	0.826
Province fixed	√	√	√	√	√	√
Time fixed	√	√	√	√	√	√
Sample size	77	77	33	33	33	33

Note:

*  p < 0.05 and

**p < 0.01 indicate significance at the 5% and 1% levels, respectively, with t values in parentheses. The same annotations are used in the following table.

To ensure the robustness of the findings, an annual-scale interval regression is used for further testing. In Columns (3) and (4), the time range is fixed to 2016–2018, and in Columns (5) and (6), the time range is adjusted to 2018–2022. The regression analysis in two different periods yields consistent results, and the effects of new qualitative productivity forces on the quality-effective change in the sports industry are significant at the 0.01 level. Moreover, the regression coefficients are all positive, and the results support H1.

### Tests for mediating effects

To investigate whether new qualitative productivity forces can indirectly promote quality-efficiency change in the sports industry through industry‒university‒research cooperation, the stratified regression method is used to test the mediating effect model. First, we observe whether Parameter a1 in Equation (11) is significant; if it is significant, we proceed to validation. Then, we test the significance of β1 and γ2 in Equations (12) and (13); if they are both significant, there is a mediating effect. Finally, we test the significance of γ1 in Equation (13); if it is significant, then part of the mediating effect is confirmed, and if it is insignificant, the mediating effect is full. The regression results are shown in Table 5.

**Table 5:** Mediating effects test.

Variant	Shd (1)	Cw (2)	Shd (3)
Nqp	1.713** (6.301)	0.436 * (2.256)	1.567** (5.705)
Cw			0.334 * (2.104)
Control variable	√	√	√
Constant	‒0.311** (‒2.912)	0.604** (7.949)	‒0.513** (‒3.616)
R^2^	0.346		0.383
Province fixed	√	√	√
Time fixed	√	√	√
Sample size	77	77	77

As shown in Table 5, the regression coefficients of the development level of new qualitative productivity forces in Models (1) and (2) are 1.713 and 0.436, respectively, and both reach a significant level, which indicates that new qualitative productivity forces not only have a significant positive effect on the quality‒performance change in the sports industry but also have a significant effect on promoting the level of industry–university–research cooperation. The coefficients of new qualitative productivity forces and industry–university–research cooperation in Model (3) are 1.567 and 0.334, respectively, and both reach a significant level, which indicates that new qualitative productivity forces can indirectly promote quality-effective change in the sports industry and improve the intensity of industry–university–research cooperation. Additionally, a part of the mediating effect is established, and H2 is supported. Therefore, to accelerate quality-effect change in the sports industry and realize its advanced development, all regions should continually improve the intensity of cooperation between industry, academia and research; strengthen the complementary optimization of the resources and functions of sports enterprises, sports colleges and universities and sports research institutes; and provide support for new qualitative productivity forces to increase the quality and efficiency of the sports industry.

### Regional heterogeneity tests

As Table 6 reveals, the regression coefficients of the impact of new qualitative productivity forces on quality-effective change in the sports industry are 0.719 in the upstream region, 1.545 in the midstream region and 1.269 in the downstream region, and these results are positive and significant in the midstream and downstream regions. These results indicate that improvements in new qualitative productivity forces in these regions significantly contribute to the regional level of quality-effective change in the sports industry. Conversely, the effect is not significant in the upstream region. Overall, H2 is valid; that is, new qualitative productivity forces promote quality-effective change in the sports industry, and this influence is characterized by regional heterogeneity. The possible reasons for this heterogeneity are as follows. First, midstream and downstream regions usually have higher levels of economic development and more mature industrial structures than upstream regions do, making the former more conducive to the rapid achievement of new qualitative productivity forces. Improvements in new qualitative productivity forces, such as those realized through technological innovation and talent concentration, can directly increase the quality of sports industry development and enhance industrial efficiency and competitiveness. In contrast, upstream regions may face problems related to singular industrial structures and a relatively weak economic foundation, and the direct promotion of the sports industry through improved new qualitative productivity forces is limited; this effect must be cultivated and structurally optimized over a longer period before it can be manifested. Second, midstream and downstream regions tend to benefit from better policy support—such as through financial support, tax incentives and talent cultivation and introduction policies—and market environments than upstream regions do. These factors are conducive to the cultivation and development of new-quality productive forces and provide good conditions for quality-effective change in the sports industry. However, upstream regions may have deficiencies in terms of policy support and the market environment, thus limiting new qualitative productivity forces and quality-effective change in the sports industry. Third, different regions are characterized by distinct cultures and consumption habits, which directly affect the mode and path of quality-effective change in the sports industry. The midstream and downstream regions may pay more attention to the cultivation of new types of sports consumption and invest heavily in funding for new product development, which is conducive to the expansion and extension of the sports industry chain. Upstream areas may have low enterprise digitization levels due to the limitations of culture and consumption habits and are subject to certain constraints in the digital transformation of the sports industry, thereby making it difficult to promote new qualitative productivity forces.

**Table 6:** Regional heterogeneity regression.

Variant	Upstream region (Shd)	Midstream region (Shd)	Downstream region (Shd)
Nqp	0.719 (1.940)	1.545^**^ (4.534)	1.269^**^ (3.385)
Pgdp	-0.034 (-0.318)	0.521^*^ (2.334)	2.583^**^ (5.264)
Mark	0.313 (1.302)	-0.736 (-0.975)	0.155 (0.677)
Tec	0.138^**^ (3.082)	0.002 (0.039)	0.184 (1.961)
Ind	‒0.625 (‒1.204)	‒0.561 (‒1.586)	‒0.000^**^ (‒4.065)
Gover	1.227^*^ (2.213)	1.037 (0.953)	‒0.854 (‒1.881)
Open	0.330 (0.975)	0.894 (1.260)	‒1.961 (‒1.335)
Constant	‒1.850 (‒1.682)	‒3.939^*^ (‒2.161)	‒29.123^**^ (‒5.613)
R^2^	0.892	0.442	0.970
Province fixed	√	√	√
Time fixed	√	√	√
Sample size	28	28	21

## Conclusions and recommendations

### Conclusions

(1) New qualitative productivity forces can effectively and directly drive quality-effective change in the sports industry regardless of whether other control variables are considered. Among the effects of the control variables on quality-effective change in the sports industry, economic development, marketization, technological innovation, industrial structure, and opening up to the outside world all have significant positive effects on quality-effective change in the sports industry, whereas the degree of government intervention has a significant negative effect on quality-effective change in the sports industry.
(2) New qualitative productivity forces can indirectly promote quality-effective change in the sports industry through industry–university–research cooperation. The results of the mediating effects test confirmed that industry–university–research cooperation plays a partial intermediary role in promoting quality-effective change in the sports industry through enhanced new qualitative productivity forces.
(3) Regional heterogeneity exists in quality-performance change in the sports industry driven by new qualitative productivity forces. The midstream and downstream regions of the Yangtze River have a significant positive effect on quality-performance change in the regional sports industry, whereas the upstream regions do not have any obvious effect. Because of faster economic development, a strong industrial structure, favorable policy support and a market environment in the midstream and downstream regions of the Yangtze River, new qualitative productivity forces can be more effectively transformed into driving forces for improving the quality and efficiency of the sports industry, whereas the effect of new qualitative productivity forces in the upstream region in promoting quality-effective change in the sports industry has not yet been fully manifested.

### Recommendations

(1) Focus on fostering new-quality industries and strengthening their application in the sports industry. The results of this study show that new qualitative productivity forces have a significant positive effect on quality-effective change in the sports industry. New qualitative productivity forces are advanced and align with the new development concept, and their value relies mainly on new-quality industries, including emerging and future ones. Therefore, the important role of emerging and future industries in the development of the sports industry should be clarified, and their application in various fields of the sports industry should be increased. Artificial intelligence and big data can be used to increase the intelligence level of the sports industry and accelerate the digital transformation and upgrading of the sports industry. Furthermore, the combination of biotechnology and the sports industry can be explored, the fusion and development of the information technology and sports industries can be strengthened, the exchange and cooperation between new-quality and sports industries can be promoted, and specific measures for the application of emerging and future industries in the field of sports can be constantly improved.
(2) Emphasize the intermediary effect of cooperation among industries, universities and research institutes to promote quality-effective change in the sports industry. Research shows that through industry–university–research cooperation, new qualitative productivity forces can indirectly promote quality-effective change in the sports industry. Therefore, it is necessary to accelerate the standardization and networking of industry–university–research cooperation to help the sports industry improve quality and efficiency. Standards and norms for industry–university–research cooperation in sports should be developed and widely publicized; the cooperation process among all parties should be clarified, as should their responsibilities, rights and obligations; a demonstration area for standard promotion should be established, the effectiveness of standards should be demonstrated through applications, the influences and driving forces of the standards should be clarified, a mechanism should be established for monitoring and evaluating the implementation of the standards for industry–university–research cooperation, cooperative projects should be regularly evaluated, and the standard system should be adjusted and improved in a timely manner according to the results. Activities such as virtual meetings for cooperative projects, technical exchange meetings, seminars and symposiums can promote the exchange of information and combination of ideas among the sports industry, academics and research institutes. Additionally, cooperative networks involving industries, academic entities and research institutes can be formed; geographic and industrial barriers can be overcome; and enterprises related to the sports industry, universities and research institutes in different regions and industries can be encouraged to cooperate extensively and in depth.
(3) Implement locally adapted strategies to develop new qualitative productivity forces in sports. Research has revealed spatial differences in the role of new qualitative productivity forces in the process of promoting quality-effective change in the sports industry. Therefore, local governments should conduct comprehensive analyses of sports market demand, sports innovation technology, sports human resources and natural resources in accordance with regional characteristics and propose a set of optimal programs that are suitable for enhancing the quality and efficiency of the local sports industry alongside its transformation and development. The central government should formulate policies for different regions. For regions with strong sports market demand but high labor and land costs, the construction of a new system of resource-saving service industries should be accelerated; for regions with weak innovative technologies but abundant resources, the support for the technological innovation of sports enterprises in the region should increase. Based on local conditions, the top-level design should be strengthened, the industrial layout should be continually optimized, new qualitative productivity forces in sports should be cultivated and developed, and the synergistic development of the regional sports industry should be promoted.

## Supporting information

S1 Raw data Sports Industry-New Qualitative Productivity Forces-Mediator and control variable raw data. (ZIP)
